# Rumination as a Mediator of Chronic Stress Effects on Hypertension: A Causal Model

**DOI:** 10.1155/2012/453465

**Published:** 2012-02-16

**Authors:** William Gerin, Matthew J. Zawadzki, Jos F. Brosschot, Julian F. Thayer, Nicholas J. S. Christenfeld, Tavis S. Campbell, Joshua M. Smyth

**Affiliations:** ^1^Department of Biobehavioral Health, The Pennsylvania State University, 315 HHD East, University Park, PA 16802, USA; ^2^Clinical Health and Neuropsychology Unit, Institute of Psychology, Leiden University, Leiden, The Netherlands; ^3^Department of Psychology, The Ohio State University, Columbus, OH 43210, USA; ^4^Department of Psychology, University of California San Diego, La Jolla, CA 92093, USA; ^5^Department of Psychology, University of Calgary, Calgary, AB, Canada T2N 1N4

## Abstract

Chronic stress has been linked to hypertension, but the underlying mechanisms remain poorly specified. We suggest that chronic stress poses a risk for hypertension through repeated occurrence of acute stressors (often stemming from the chronic stress context) that cause activation of stress-mediating physiological systems. Previous models have often focused on the magnitude of the acute physiological response as a risk factor; we attempt to extend this to address the issue of *duration of exposure*. Key to our model is the notion that these acute stressors can emerge not only in response to stressors present in the environment, but also to mental representations of those (or other) stressors. Consequently, although the experience of any given stressor may be brief, a stressor often results in a constellation of negative cognitions and emotions that form a mental representation of the stressor. Ruminating about this mental representation of the stressful event can cause autonomic activation similar to that observed in response to the original incident, and may occur and persist long after the event itself has ended. Thus, rumination helps explain how chronic stress causes repeated (acute) activation of one's stress-mediating physiological systems, the effects of which accumulate over time, resulting in hypertension risk.

## 1. Introduction

The question of why one person develops high blood pressure (BP) and another does not has long been controversial. For example, Freud in the 1930s hypothesized that keeping one's anger in will cause BP to rise. Theories of stress and disease came a bit later, and a multitude of experimental and field studies show that stress is indeed linked, both epidemiologically and causally, to physiological dysregulation and chronic illness. The model we present here builds on this research but attempts to take it a step further. 

### 1.1. Stress as a Risk Factor for Hypertension

It is welldocumented that “stress” (broadly and variously defined) is a risk factor for hypertension (HTN) and other cardiovascular disease (CVD), including events and all-cause mortality [1–8]. The mechanisms that underlie this association, however, have not been as clearly specified. This is due, in part, to the ambiguity concerning the nature of “stress.” The construct has been used and measured in many different ways, with little standardization across studies. Moreover, the notions of “chronic” and “acute” stress, and how they relate to one another, have not been clearly delineated, with little theoretical work targeted at identifying the respective domains of each. To the extent that we believe that stress—however it is defined—is indeed a risk factor for HTN, it is crucial that we identify the pathways through which it may lead to sustained elevated BP if we are to develop effective interventions to reduce stress, with the aim of reducing high BP and associated long term disease risk. In this paper, we will attempt to provide specifications concerning the respective domains of acute and chronic stress and to present a model and review evidence that points to *rumination*—the cognitions and negative affect that stem from the experience of an acute stressor—as a key mechanism through which stress exerts its pejorative effects on autonomic functioning and the resting BP level. We then discuss implications for interventions and future research.

### 1.2. Acute Stress

In this paper, we operationalize acute stress as a situational factor. That is, a discrete event that stems from some aspect of one's immediate situation, and, for the most part, has an impact on the person only to the extent that (1) it is perceived, (2) the perception leads to negative cognitions and affect, and (3) that these cause perturbations in the various physiological stress-mediating systems (PSMSs) including autonomic, hypothalamic-pituitary-adrenal (HPA), and immune systems. By this definition, an acute stressor may also arise internally (i.e., a mental representation) and provoke continuing thoughts and emotions, and, concomitantly, their effects on the autonomic and other PSMSs. We have noted “for the most part” as some acute stressors will have effects independent of this mechanism. For example, a pin-prick may cause such perturbations, but via different, more direct, channels not involving cognition. In this paper, we focus on those acute stressors that exert their effects on the PSMSs via the ruminative (perception-negative cognition/affect) pathway. In research examining stress effects on BP, acute stressors have mostly been defined as standardized laboratory tasks such as mental arithmetic or the cold pressor. We and others have used an anger recall task, which we have shown is similarly effective in raising the BP and HR, decreasing heart rate variability (HRV), increasing cortisol, and other outcomes including impaired endothelial function [9–13].

### 1.3. Chronic Stress

We do not currently have a fully useful definition and circumscribed domain for chronic stress; in the literature, it often appears to be a case of “we know it when we see it”. Thus, caregiver status, low socioeconomic status (SES), an unpleasant low-paying job, and a stint in jail—all these seem like obvious sources of chronic stress. But what are the defining characteristics? Certainly, there is a sense that part of it is the “ongoingness” of the situation and part is the pervasiveness of the situation. Thus, one may find oneself continuously in the midst of the situation—being in prison is an extreme example, finding oneself further and further in debt with creditors calling at all hours is another. It is not necessarily the case, however, that even under such pervasive and ongoing conditions, one is continuously attending to the situation. Thus, the individual instances or moments that *do* invoke the situation and do provoke a reaction—the creditor's phone call, for example, or a worry episode regarding mounting dept—are intermittent, with a greater or lesser frequency depending on the nature of the chronic stressor. And we would regard the individual instances as examples of acute stressors, per the previous section, that arise from (are potentiated by) the chronically stressful context.

For the purposes of this paper, as with acute stress, we define chronic stress as a situational variable. In this instance, however, “situational” refers to a “background” that increases the likelihood that acute stressors—relating to the chronic stress situation—arise. (Acute stressors may also, of course, arise from sources not related to one's chronic stress). *Thus, we propose that chronic stress matters largely to the extent that it consists of a background situation that gives rise to acute stressors, with these acute stressors having an impact on the PSMSs.* It is important to note that this chronically stressful situation may affect one's BP via other pathways; thus, chronic stress has effects beyond “merely” being the source of acute stressors. For example, the parental caretaker may begin eating poorly and gain weight, and may increase salt intake, each of which will affect the BP via other pathways (i.e., health-related behaviors, in this instance, although these may arise in response to both the chronic stress background and the acute stressors that may arise).

So far, we have stated that acute stressors arise from environmental conditions, and often more frequently when the environmental conditions are characterized as chronically stressful. An individual exposed to such a situation will likely be exposed to “reminders” of the situation—such as the creditor's phone call—and may be apt to periodically recall the terrible conditions even without environmental cues. We suggest that there is functionally no differentiation between these “reminders” and the effects of the acute stressors, both produce acute stress, insofar as each leads to negative cognitions and affect and perturbations in the PSMSs. Posttraumatic stress disorder (PTSD) offers an example of this, albeit an extreme one. In some cases, a single event, even a brief one, may lead to persistent effects on cognition and affect. These effects can persist for decades as individuals with PTSD continue to deal with intrusive thoughts and emotions that recreate representations of the event (and associated stress and other physiological responses). In fact, evidence suggests that the frequency of these reexposures to the original event is strongly related to PTSD severity [14].

## 2. Rumination

A limitation of most acute stress models is that they do not take into account the *duration of exposure*. Rather, the focus is solely on the *magnitude* of the acute response. Thus, a person that exhibits a larger BP response to an acute stressor is presumed to be at greater risk for HTN than one who exhibits smaller responses. Stressor-related thoughts that may emerge over time as a result of (but temporally distal from) the acute stressor can also cause acute elevations in the PSMSs. Moreover, these physiological responses may persist as long as the recurring cognitions/emotions (e.g., anger or anxiety) persist. Relevant to this, Glynn and colleagues [15] have shown that recall of a laboratory stressor to which the subject had been exposed in a previous session produced BP responses comparable to those which occurred when the participant was exposed to the stressor itself. Thus, the cognitive and affective sequelae of the acute stressor may have as great an effect on the PSMSs as the original initiating event, long after that event has occurred (with the limits on the ruminative responses likely only constrained by the verisimilitude and intensity of the internal representation).

We thus broadly define rumination as *affect-laden cognitions that (1) result from exposure to an acute stressor (external in the environment or an internally generated representation); (2) in most individuals cause acute activation of relevant physiological stress-mediation systems; (3) “outlast” the original stimulus/acute stressor (i.e., the ruminative thoughts and accompanying negative affect*—*and their effect on physiological activation—persist even after the stressor itself is no longer present); (4) often reemerge*—*in some, over quite long periods of time; (5) typically do not lead to productive solutions, but rather resemble an “endless loop”, that does not lead to insight or resolution of the issue. *We also note that, in addition to the term “rumination”, there are many other related constructs. Some are often used interchangeably, such as perseverative cognitions, whereas others imply some differences. For example, distinct from rumination, which typically concerns *past* stressful events, one may experience anticipatory negative cognitions and affect concerning *future* events, which are often termed worry or anxiety; these states, too, can produce sustained effects on the PSMSs. It is as yet unclear whether these are distinctions that make a difference in terms of physiological consequences and/or HTN risk.

**Figure 1:**
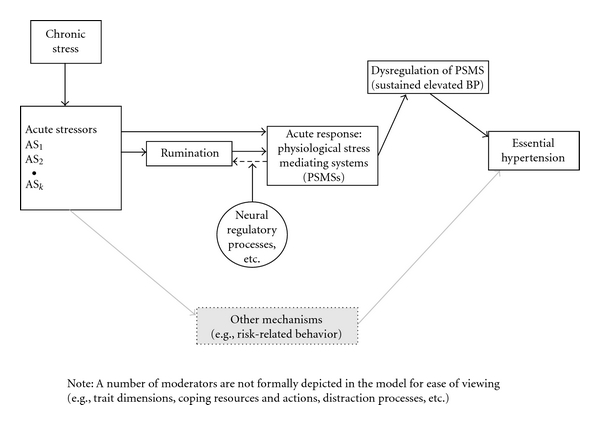
Schematic of the “chronic stress—rumination—HTN” model.

We thus propose that it is largely the cumulative *duration of exposure* to this persistent representation of the acute stressor, with its attendant effects on, and eventual potential dysregulation of the stress-mediating physiological systems that contribute to sustained elevated BP and to HTN. That is, “actual” stressors are important, but that the ruminative representations (and their consequences) contribute more to observed PSMSs responses and to HTN risk.  Figure 1 shows a schematic of a model that illustrates these predictions and pathways.

### 2.1. The Proposed Model

Figure 1 outlines the conceptual model. Chronic stress (a situational factor) sets the stage for and gives rise to the occurrence of acute stressor incidents that, depending on their nature, may lead directly to activation of the PSMSs (e.g., a pinprick) or may lead to ruminative thoughts and emotions that in turn may produce PSMSs responses. Coping resources are proposed as a moderator of the acute stressor—rumination link that in part determines whether the incident produces a ruminative response. Thus, many variables that have been implicated in stress effects are accounted for *before* they are perceived as stressful; and *rumination* is affected by the composite as a whole. For example, the perceived availability of social support operates before appraisal and thus, in some individuals, reduces the likelihood that the stimulus will activate ruminative thoughts and affect.

The thoughts and emotions that are evoked by the acute stressor affects the PSMSs, causing acute perturbations in the various systems. In those who tend to ruminate (more frequently and/or for longer duration), the effects on the PSMSs will be prolonged, and thus, *the individual is exposed to the effects of the stressor for a more sustained period of time*, which may lead to a resetting of homeostatic set points, including elevated resting BP. Herein, lies the critical role of *duration*, which many models of acute stress tend to ignore.

The dashed arrow shows a feedback mechanism such that the autonomic activation that results as a function of rumination itself becomes a stimulus that potentiates the negative emotions and cognitions; these in turn maintain the autonomic activation, and so on. Why should this feedback loop ever stop? The person may fall asleep, or may become distracted—we view “distraction” as a potential moderator of this loop—leading to termination of the rumination and its effects on the PSMSs when a distractor of sufficient potency to “drive out” the ruminative thoughts and replace them with others is encountered. We also show a moderating effect of neural inhibition by the prefrontal cortex on the association between acute stress and rumination/worry. Finally, a number of other pathways known to exist, but not addressed as central to this paper, are depicted in greyscale (as rumination is not the only pathway by which stress may affect HTN). Specifically, addressing these additional pathways is, however, beyond the scope of this paper. Circles indicate that the variable is proposed as a moderator.

### 2.2. Old Wine in New Bottles?

One issue is whether the proposed model represents a mere restatement of existing theoretical formulations or offers practical and/or theoretical advance. We are aware that many of the definitions and relationships among constructs herein have been proposed and discussed by others, many of whom developed theories that had a major impact on how stress was conceptualized. Thus, the models proposed by Selye [16] and by Lazarus and Folkman [17] are progenitors of the current proposed model. There are, as well, many other theories that address aspects of the questions at issue, and we have borrowed from some of these. The model as we present it, however, has been informed by recent advances in the psychophysiological, health, and social psychological literatures, and addresses issues not formerly considered. Moreover, this model is not intended to propose a general theory of stress; rather, the goal is to understand the factors that lead chronic stress to be a substantial risk factor for HTN, and the model we present focuses on this problem and the central role of rumination.

### 2.3. Unconscious Rumination

Although we have been discussing rumination as a largely active process, data from several lines of research suggest that individuals may be unaware of some portion—potentially a large portion—of their stress-related cognitions. Yet, even these “unconscious thoughts and feelings” can have an activating effect on the autonomic and other stress-mediating physiological systems. (We operationalize the term “unconscious” as indicating only that it occurs while one's attention is not or cannot be directed toward it, or is directed elsewhere, that is, out of awareness.) There is a rich body of research converging on the idea that “implicit emotion” plays a role in mental and psychosomatic disorders [18]. In the last two decades, developments in cognitive and social psychology have strongly indicated that a great deal of cognition occurs at the unconscious (or automatic) level [19, 20]. To date, several studies have shown that neurophysiological responses occur in response to threatening information that is shown subliminally, an accepted experimental model of “unconscious cognition” [18, 21]. More importantly for this paper, it has also been shown that such subliminal stimuli can increase BP [22–24].

Regarding chronic stress, we have found that (conscious) rumination was linked to cardiac activity in daily life, and this activity persisted for hours after the rumination itself had ended [25]. We and others [26–29] also found effects of daily stress and rumination on cardiovascular activity during subsequent sleeping at night, during which conscious rumination is presumably not possible. On the basis of these findings, some researchers [21, 30] have recently proposed that stress research may have missed an essential phenomenon by focusing solely on the conscious “tip of the iceberg” of stress-related thoughts and feelings. Unconscious rumination may be responsible for a considerable portion of cardiovascular activity in one's daily life. Pending development of appropriate measures—which remains a challenge—studying unconscious stress in the context of HTN and other stress-related somatic conditions may turn out to be a particularly fruitful pathway for future investigation.

### 2.4. Neurovisceral Moderators of Rumination

The perseverative cognitions that characterize rumination are thought to be under tonic inhibitory control by the prefrontal cortex [31, 32]. There are several conditions, including chronic stress or having an anxiety disorder, that lower prefrontal inhibition and thus render one more vulnerable to rumination as well as to prolonged and indiscriminate responses to environmental stimuli. Low prefrontal inhibition is characterized by low parasympathetic activation, which can be measured by low heart rate variability (HRV). (For a more detailed explanation, see [33, 34].) Low tonic levels of HRV might indicate a predisposition to “err on the side of caution” when confronted with threat. As such, an excitatory *positive feedback loop* is allowed to emerge, reflected in the psychological level in hypervigilance and rumination. As a consequence, the normally fine-tuned ability to adjust to changing environmental factors becomes a relatively rigid, inflexible response disposition, which is in fact a continuation of the default defense response in the absence of clear threat signals. This is reflected by a failure to recognize safe/neutral environmental signals and by responding to them as if they are threatening. In support of this idea, patients suffering from generalized anxiety disorder have been shown to have lower tonic levels of HRV, when compared to nonanxious controls [35]. Furthermore, people with low HRV have been shown to have an attentional bias for threatening information and interpret ambiguous situations more negatively [36].

In sum, we propose that low prefrontal inhibition, indexed by low HRV, predisposes people to respond with enhanced cognitive, affective, and physiological activity to stressors. This, in combination with the psychological vulnerability factors for rumination discussed above, can cause even neutral stimuli to trigger the stress response. As a consequence, the total time that people ruminate and worry about stressful events increases, thereby adding to the total duration of exposure to stress representations and their physiological effects in daily life.

### 2.5. Review of the Rumination Literature

The overarching prediction derived from the model is that an important mechanism underlying the observed relationship between “chronic stress” and HTN is *rumination*, which typically stems from exposure to an acute stressor. *Thus, compared to individuals who show a lower tendency to regulate their angry and anxious and dysphoric thoughts using ruminative processes, high “trait” ruminators should be more likely to have more frequent, intense, and longer-lasting, ruminative thoughts and emotions as well as more (future) reoccurrences and occurrences that persist longer into the future.* In support of this prediction, rumination has been shown to increase engagement in depressed thinking [37, 38], is related to negative emotions, including anxiety [39, 40], anger [41–43], and depressed mood [43–45], and can prolong negative affect [42, 46]. Rumination appears to be a relatively stable characteristic, as test-retest is high for both anger [42] and depressed rumination scales [47, 48] (for periods ranging from one month to one year). Tendency to ruminate also predicts future occasions on which rumination is likely to occur. For example, ruminators were more likely to experience a depressive episode in the ensuing 18 months than nonruminators [47], with a similar result found by Nolen-Hoeksema [40]. Change in rumination levels predicted changes in depression over four months [48]. Furthermore, higher tendencies toward angry rumination were related to increases in experienced and expressed anger and to decreased satisfaction with life on future occasions [42].

#### 2.5.1. High “Trait” Ruminators Should Also Be More Likely to Exhibit the Physiological Activation that Tends to Result from Such Thoughts

Increases in rumination have consistently been linked to higher blood pressure during recovery periods [49–54]. For example, compared with nonruminators, ruminators after an anger recall incident were more likely to brood about their anger thoughts during a recovery period in which they were not distracted [49]. Importantly, this tendency to ruminate translated-to-slower blood pressure recovery for these ruminators, demonstrating the ability for rumination to sustain high blood pressure. Other studies support this view as well; participants induced to recall a stressor they experienced in the lab (i.e., ruminate about the stressor) showed increases in blood pressure regardless of whether they ruminated about the stressor 30 minutes after it happened or one week later [50]. In other words, the mental representation of the stressor increased blood pressure regardless of temporal “distance” from the original event. Importantly, the relationship between rumination and blood pressure is not just in the moment. Compared to nonruminators, individuals who ruminate have higher resting [55, 56] and ambulatory blood pressures [56–58]. For example, Ottaviani and colleagues [58] had participants engaged in an anger recall interview, which successfully raised blood pressure. Next, half of the participants were assigned to a distraction condition where rumination was abolished, while the other half was not distracted and were allowed to continue ruminating about the anger inducing incident. Pertinently, not only did this nondistracted, ruminating group have elevated blood pressure during the recovery period immediately after the anger recall, but their blood pressure remained significantly elevated compared to their baseline levels when examining their mean ambulatory blood pressure readings taken over the ensuing 24 hours. Thus, there is evidence demonstrating the power of rumination to sustain elevated blood pressure not just over minutes, but hours and days.

#### 2.5.2. High “Trait” Ruminators Should Tend to Exhibit Dysregulation of the Physiological Stress-Mediating Systems

Higher blood pressure tends to beget yet higher blood pressure over time [59]. In strictly biophysical terms, one mechanism by which the blood vessels in the body regulate blood flow is by constricting and expanding. As blood pressure increases, either more blood (i.e., higher cardiac output) enters the blood vessels or the blood enters the vessels with greater force (i.e., mean arterial pressure); either way, the blood vessels must expand and contract more to regulate blood flow. Over time, this process increases the thickness of the muscle around the vessels—the more often a person has higher blood pressure, the more the vessels work and the faster and thicker this layer grows. These thicker arterial walls are, however, more resistant to the force of blood flow, requiring the heart to work harder to pump enough blood hard enough for the vessels to properly regulate. With greater effort from the heart, blood pressure goes up, which further increases the muscles around the arteries.

This process explains how having sustained, untreated HTN in one's life is an independent predictor of cardiovascular morbidity [60]. High blood pressure can thus cause irreversible structural changes to the body. This process is conceptually similar to that of allostatic load [61], which can be described as a resetting of the physiological set points in an attempt to maintain homeostasis and thus function, but at a new set point (e.g., higher blood pressure). In the short-run, achieving allostasis is adaptive (e.g., the higher blood pressure allows the blood vessels to continue to regulate blood flow), but in the long term can have quite negative consequences [62]. Importantly, and supportive of the model presented here, the stressors that cause allostatic load are not major, impactful (but infrequent) stressors, but rather the cumulative effect of more frequent minor psychosocial stressors [63]. Key to this theory is that these frequent, more minor stressors have to produce sufficiently long response durations so as to lead to allostasis [64]. As discussed, the presence of an initial stressor does not automatically lead to the prolonged activation that causes the resetting of the body's set point or allostasis [65]. Rather, it is the presence and persistence of the cognitive representations (i.e., rumination, worry, and other perseverative cognitions) that can later reactivate/recreate a stressor in one's mind; this process and the resultant physiological changes are the circumstances that characterize and explain the chronic stress-HTN relationship.

## 3. Potential Factors Not Shown in the Model

We are attempting to describe a highly complex process and to understand why some are prone to ruminate and others are not, how attention—clearly a key determinant of the nature of one's conscious thoughts—as well as signal detection and hypervigilance for perceived threats and insults is implicated in the process. Finally, we note that classical conditioning processes undoubtedly play a role, for example, a conditioned response to an eliciting stimulus may evoke the cognitive and affective response to past unconditioned aversive events. Consideration of these factors is beyond the scope of this paper, but will need to be addressed as our understanding of the processes increases.

### 3.1. A Brief Note Concerning Applications of the Model for Interventions to Reduce Stress and Thus Lower Risk for HTN and CVD

There are many avenues of approach to such interventions, in part because there are several pathways by which stress may cause HTN and heart disease. Thus, obvious targets are stress-related behaviors that may increase one's risk, such as smoking, weight gain, lack of exercise, increased salt intake, and interpersonal behaviors that may cause subsequent and ongoing stressful incidents, and so on. Interventions may, and do, target coping behaviors and skills. The model we propose suggests that an additional useful point of intervention may be the ruminative processes that can convert acutely stressful experiences into recurrent or chronic stress. For example, one approach would be the cognitive reframing of stress-related negative thoughts and affect as a means of undermining their power to evoke subsequent negative thoughts and affect, and thus reducing the duration of exposure to sustained activation of the PSMSs. Even here, several strategies suggest themselves. Cognitive behavior therapy [CBT] specifically targets such cognitions, and is a well-validated clinical technique. One large clinical trial—ENRICHD [66]—has indeed used CBT as an intervention to reduce CVD risk (in conjunction with a social support manipulation), but the results were largely negative. Rumination was not measured in that study, however, we would suspect that if the frequency and severity of the negative thoughts and cognitions were not reduced, such an intervention would be unlikely effective. Clinicians have developed interventions to train people to learn to forgive past perceived transgressions, and others have targeted one's ability to “let go” of anger. Although intriguing, their effects on rumination and on PSMSs activation have been the subject of only a handful of studies and thus data are sparse for these approaches [67]. We hope this model spurs additional intervention work that tests a range of intervention targets, potential mediators, and a wider range of outcomes. On a final note, we suggest that a combination of procedures, likely targeting behavior as well as cognition and affect, may have better results than either alone.

## 4. Summary and Conclusions

Defining “chronic stress” and understanding the pathways by which it may contribute to sustained elevated BP and eventual HTN is a large undertaking and certainly involves more than one mechanism. We have addressed one pathway, but others, including changes in risk-related behaviors and in the direct effects of chronic stress on HTN, are not addressed here, nor are physiological BP regulatory systems including the angiotensin system, short term regulatory systems including the baroreflex, or the role of endogenous opioids. To eventually form a comprehensive model of the development and maintenance of pathogenically high-sustained BP resting level, these pieces must be addressed as well. Our model attempts to make a more modest and circumscribed contribution, but we hope to integrate these potential mediators and moderators into the model in future studies.

We propose that acute BP and other changes in response to experiencing an acute stressor may be less important than the continuing PSMSs activation that often persists after the incident itself has long ended and may reoccur when the memory of the stressor is activated. Robert Sapolsky addressed this issue in his book *Why Zebras Do not Get Ulcers* [59], in which he noted in his evocative example that zebras do not have the capacity to recall the stress (the failed attack from the lion, e.g.,) that has beset them; therefore, they neither ruminate about the past nor worry about the future, and thus do not suffer the PSMSs consequences of engaging in such mental activity. And thus do not suffer ulcers or, presumably, HTN, at least not due to the pathway proposed in this paper. (The supposition here is that these acute events occur only intermittently for the beleaguered zebra; if they are occurring many times a day over long periods of time, the distinction between acute and chronic stress becomes blurred). It also may be worth speculating about the evolutionary underpinnings. For some time, evolution has been shaping and perfecting the “fight-or-flight” response system that is designed to engage under acute threat. Awareness, or consciousness, however, emerged only a short time ago in evolutionary terms, and it was not, according to our model, until this point that mammals—humans, at least, and perhaps some infrahumans—developed the capacity to self-reflect and, thus, to ruminate. Therefore, zebras do not develop ulcers, but humans do (as well as elevated BP and HTN risk).

It is widely accepted that chronic stress must be filtered in some manner by sensation and—more importantly—perceptual processes, thus leading to individual differences in response to environmental challenges or stressors. Similarly, the role of duration of exposure to the stress—or, as we suggest, to the contents of one's thoughts and emotions—*is*, we argue, the representation of the chronic stress in its purest form; it is, after all, those thoughts and emotions that largely affect the stress mediating physiology, which itself is a more proximal cause of HTN and CVD. Do the data support the model? As reviewed herein, parts of the model have been tested in different laboratories, often using different approaches, and the results seem to hang together to suggest that indeed, rumination is one important pathway by which the effects of chronic stress on the development of HTN are transmitted. Other portions of the model remain to be more systematically evaluated. Scores of studies, however, conducted across several laboratories, have turned their attention to the role of rumination and perseverative cognition, and we anticipate that many of the blanks will be filled in as research on this topic progresses. One important line of inquiry is to apply more intensive, within-person data capture approaches that can carefully document the temporal dynamics of these processes (such as the initial occurrence and response to an acute stressor, and then its reoccurrence via ruminative processes and their associated responses; see [68]). Coupling ambulatory psychophysiology (e.g., BP) with ecological momentary assessment techniques is one well-validated approach that holds great promise in this regard [69].

We believe that the model we have proposed represents an advance in the manner in which we conceptualize stress and its effects on blood pressure and cardiovascular disease. Partly, this is because of the basis it provides to develop useful interventions as well as precise targets of those interventions. Researchers have been ruminating about the nature of stress for some time; we hope that this research will help turn these ruminations into productive ideas for further exploration.
